# Dopaminergic restoration of prefrontal cortico-putaminal network in gene therapy for aromatic l-amino acid decarboxylase deficiency

**DOI:** 10.1093/braincomms/fcab078

**Published:** 2021-04-15

**Authors:** Yoshiyuki Onuki, Sayaka Ono, Takeshi Nakajima, Karin Kojima, Naoyuki Taga, Takahiro Ikeda, Mari Kuwajima, Yoshie Kurokawa, Mitsuhiro Kato, Kensuke Kawai, Hitoshi Osaka, Toshihiko Sato, Shin-ichi Muramatsu, Takanori Yamagata

**Affiliations:** 1 Department of Neurosurgery, Jichi Medical University, Tochigi 329-0498, Japan; 2 Department of Neurology, Saiseikai Kurihashi Hospital, Saitama 349-1105, Japan; 3 Department of Pediatrics, Jichi Medical University, Tochigi 329-0498, Japan; 4 Department of Anesthesiology and Critical Care Medicine, Division of Anesthesiology, Jichi Medical University, Tochigi 329-0498, Japan; 5 Department of Pediatrics, Showa University School of Medicine, Tokyo 142-8666, Japan; 6 Department of Pediatrics, Yamagata University Faculty of Medicine, Yamagata 990-9585, Japan; 7 Utsunomiya Central Clinic, Tochigi 321-0112, Japan; 8 Division of Neurological Gene Therapy, Jichi Medical University, Tochigi 329-0498, Japan; 9 Center for Gene & Cell Therapy, The Institute of Medical Science, The University of Tokyo, Tokyo 108-8639, Japan

**Keywords:** gene therapy, cortico-putaminal network, aromatic l-amino acid decarboxylase deficiency, dopamine restoration

## Abstract

Aromatic l-amino acid decarboxylase (AADC) is an essential dopamine-synthesizing enzyme. In children with AADC deficiency, the gene delivery of AADC into the putamen, which functionally interacts with cortical regions, was found to improve motor function and ameliorate dystonia. However, how the restoration of dopamine in the putamen in association with cortico-putaminal networks leads to therapeutic effects remains unclear. Here, we examined neuroimaging data of eight patients with AADC deficiency (five males and three females, age range 4–19 years) who received the AADC gene therapy of the bilateral putamen in an open-label phase 1/2 study. Using high-resolution positron emission tomography with a specific AADC tracer, 6-[^18^F]fluoro-l-*m*-tyrosine (FMT), we showed that FMT uptake increased in the broad area of the putamen over the years. Then, with the structural connectivity-based parcellation of the putaminal area, we found that motor improvement is associated with dopaminergic restoration of the putaminal area that belongs to the prefrontal cortico-putaminal network. The prefrontal area dominantly belongs to the frontoparietal control network, which contributes to cognitive-motor control function, including motor initiation and planning. The results suggest that putaminal dopamine promotes the development of an immature motor control system, particularly in the human prefrontal cortex that is primarily affected by AADC deficiency.

## Teaser

Gene therapy for Aromatic l-amino acid decarboxylase deficiency improves motor function and ameliorates dystonia. However, its underlying mechanism is unclear. Onuki et al. reported that dopaminergic restoration of the network between the putamen and prefrontal cortex in the frontoparietal control network contributes to the therapeutic effects.

## Graphical Abstract

**Graphical Abstract:**
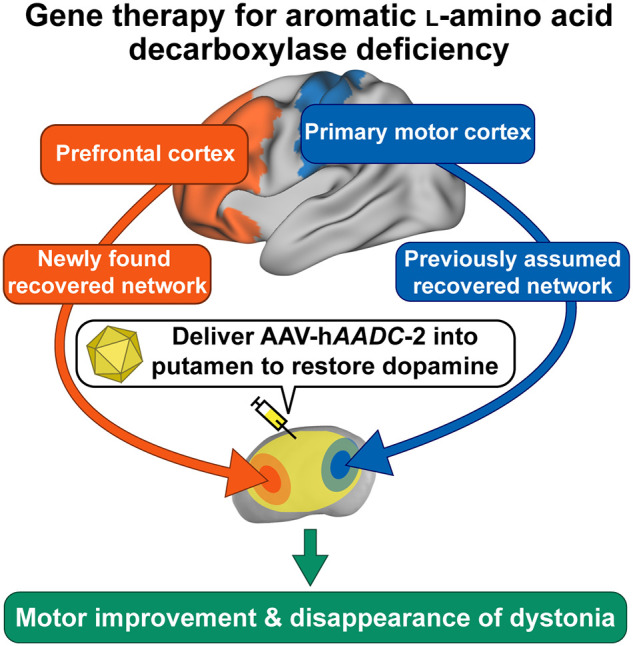

## Introduction

Aromatic l-amino acid decarboxylase (AADC) is an essential enzyme required for synthesizing dopamine, which modulates cortical inputs in the putamen. In patients with AADC deficiency (OMIM #608643), a global population of approximately 140 patients, mutations in the dopa decarboxylase (*DDC*) gene cause the profound loss of dopamine in the putamen. The primary phenotypes include developmental delay, hypotonia and movement disorders, particularly limb and truncal dystonia that results in abnormal motor control of synergistic and antagonistic muscles, and oculogyric crisis that shows a dystonic reaction of sustained upward or lateral deviations of the eyes. Recently, as a disease-modifying therapy for AADC deficiency, adeno-associated virus (AAV) vector-mediated delivery of *DDC* into the putamen was shown to provide transformative medical benefits across various patient ages, genotypes and disease severities.^1^^,^^2^ We had earlier demonstrated marked improvement of both cognitive and motor functions along with the disappearance of painful dystonia within 2 months after gene therapy and oculogyric crisis was markedly decreased in all patients.^2^

**Figure 1:**
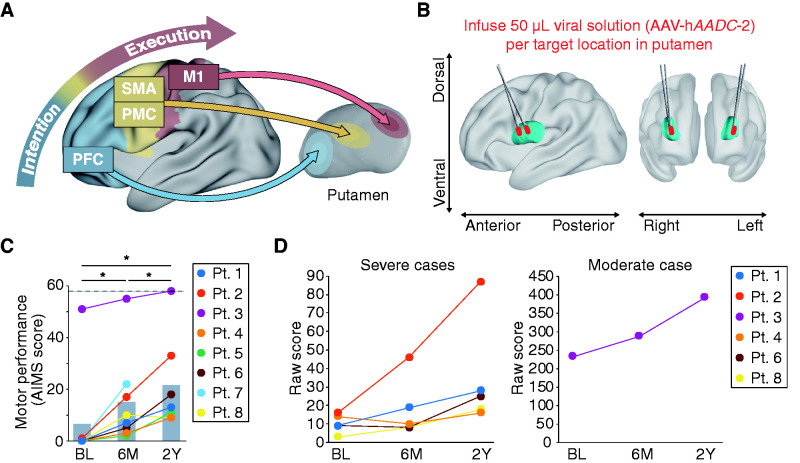
**Gene therapy for aromatic l-amino acid decarboxylase (AADC) deficiency.** (**A)** Conceptual model of the cortico-putaminal network in the motor system. (**B)** 3D model of the putamen and infusion tracks defined from a postoperative T2-weighted MR image (see Supplementary Fig. 1). (**C)** Gross motor performance scored by AIMS increased from the baseline to 2Y post-treatment. Dashed line: The maximum score of AIMS; BL: baseline; Pt: patient. **(D)** Cognition–adaptation score of the Kyoto Scale of Psychological Development showed the acquisition of new motor skills including voluntary arm extension, grabbing and holding objects, after the gene therapy. Grabbing and holding objects is recognized from 10 points, and arm extension to grab objects is recognized from 16 points.

Although dopamine is a well-known and indispensable modulator for appropriate motor performance, there is no clear information regarding the neurological mechanisms underlying the beneficial effects on the motor system after the local replacement of dopamine in the putamen. In the motor system, the cortical signals of motor intention from the prefrontal cortex (PFC) are transmitted through the premotor cortex (PMC) and the supplementary motor area (SMA) to the primary motor cortex (M1) for complex motor execution (Fig. 1A). These cortical regions participate in some networks of multiple cortical regions that functionally interact with the striatum. The functions of these networks are associated with the density of the striatal D1 dopamine receptors,^3^ spatial patterns of gene expression^4^ and resting-state activity.^5^ The subcortical regions receive these cortical inputs and send feedback outputs to the cortex, which forms a cortico-basal ganglia network. The putamen targeted by the AADC gene therapy is a hub for the cortico-basal ganglia network, which comprises functional circuits that have been implicated in various cognitive and motor processes.^6^ In fact, the putaminal area can be topographically divided into organized subsections connecting to cortical areas.^3^^,^^5^^,^^7^ It has been implicated that the therapeutic effects on the motor system are associated with the involvement of the specific AADC gene-transduced putaminal area that belongs to distinct cortico-putaminal networks. Therefore, we investigated the spatial and temporal aspects of transduction efficiency in the putamen and the association of dopaminergic intervention in the human cortico-putaminal network with therapeutic effects on the motor system after *DDC* gene delivery into the putamen in patients with AADC deficiency.

## Materials and methods

### Patients

Eight patients with AADC deficiency were enrolled in this study (five males and three females, aged 4–19 years, mean = 9.5 years, standard deviation = 5.5; see Supplementary Table 1 for the demographic features of patients). Patients 1 and 2 were siblings. The cohort included five Japanese Asian patients, two non-Japanese Asian patients and one Caucasian patient. The definitive diagnosis of AADC deficiency was based on catecholamine metabolite analysis of cerebrospinal fluid, enzyme activity and genetic components. Patients who met the following criteria were excluded: Serious illness unrelated to AADC deficiency, mild phenotype AADC deficiency with maintained locomotive ability, significant AADC enzyme activity detected by FMT-PET at baseline and unavailability of structural MRI scan. Informed consent was obtained from the parents or legal guardians of the patients. Some of the behavioural data and FMT-PET images were obtained from the same patients described previously.^2^ This study was designed as an open-label, phase 1/2 trial at Jichi Medical University Hospital (Tochigi, Japan) and was also registered with the UMIN Clinical Trials Registry (number UMIN000017802). Approval was obtained from the ethics committee for Gene Therapy at Jichi Medical University Hospital and the Science Council, Ministry of Health, Labor, and Welfare. The protocol was conducted in accordance with the Japanese Guidelines on Gene Therapy. Adaptation to the integration criteria was evaluated before each treatment, and patient status was monitored by members of the evaluation committee of the Ethical Committee for Gene Therapy at Jichi Medical University Hospital.

### Stereotactic delivery of the AAV-h*AADC*-2 vector

Clinical-grade recombinant AAV type 2 vector, AAV-h*AADC*-2, was prepared in compliance with the current Good Manufacturing Practices at Takara Bio Inc as described previously.^2^ Stereotactic surgery was performed using the Cosman–Roberts–Wells frame (Integra LifeSciences, Plainsboro, NJ, USA) to deliver the AAV-h*AADC*-2 vector into the putamen. Briefly, two target points that were sufficiently distant from each other in the dorsolateral direction, as confirmed by CT and MRI, were determined for each putamen. One burr hole was trepanned in each side of the cranial bone, and the vector was injected through a two-track insertion route. At each target point, 50 μl of the vector-containing solution was injected at a rate of 3 μl/min (a total of 200 μl containing 2 × 10^11^ vector genomes). For each injection, the needle was inserted into the deepest point of each target and then withdrawn 1 mm after each 10 μl injection. Thus, the vector was injected over a length of 5 mm at each point. All procedures were safely performed under general anaesthesia.

### Image acquisition

MRI and PET were conducted at baseline and at 6 months (6M) and 2 years (2Y) post-treatment after gene transduction. For clinical reasons, the acquisition time for 6M post-treatment is 3.5 months for patient 5. To avoid excessive motion during all imaging scans, patients received general anaesthesia. Brain structure images [T1, T2, and diffusion tensor images (DTI)] were acquired using the 3T magnetic resonance scanner (Magnetom Skyra, Siemens Healthineers, Erlangen, Germany). T1-weighted MPRAGE images were acquired with the following sequence parameters: number of slices = 224, repetition time (TR) = 1390 ms, echo time (TE) = 2.3 ms, inversion time = 800 ms, field of view (FOV) = 256 mm, flip angle = 0.85°, slice thickness = 0.85 mm, and matrix = 320 × 217. T2-weighted images were acquired by tracing the injection of the recombinant AAV type 2 vector with the following parameters: number of slices = 90, TR = 6000 ms, TE = 98 ms, FOV = 256 mm, flip angle = 150°, slice thickness = 2 mm, and matrix = 256 × 205. Diffusion-weighted images were acquired with the following parameters: number of slices = 50, TR = 8300 ms, TE = 74 ms, FOV = 256 mm, slice thickness = 3 mm, matrix size = 128 × 128, *b* value = 0 and 800 s/mm, and number of diffusion-weighted directions = 12. Combined PET and CT scanning was conducted using a PET-CT scanner (Gemini GXL, Philips, Eindhoven, The Netherlands). Patients were allowed an 80-min rest before the beginning of the emission scan session after the administration of the FMT solution of 0.12 mCi/kg in saline to an antecubital vein in patients. A CT transmission scan was obtained for attenuation correction, and subsequently, a 10-min static three-dimensional acquisition was initiated. Carbidopa, an AADC inhibitor, was not administered before the scan due to insufficient AADC activities observed in the patients. PET images were acquired with the following parameters: list-mode acquisition (LOR-RAMLA), 90 slices (slice thickness = 2 mm) covering the whole brain, FOV = 256 mm, matrix size = 128 × 128, and voxel size = 2 × 2 × 2 mm^3^. CT data were acquired for attenuation correction (140 kV, 55 mA, 5.526 s, FOV = 600 mm, matrix = 512 × 512, pitch = 0.829, and rotation time = 0.75 s). In Pt. 6 and Pt. 8 at 2Y post-treatment, the higher spatial resolution PET image was acquired using a PET-CT scanner (Vereos, Philips, Eindhoven, The Netherlands) with the following parameters: List-mode acquisition, 82 slices (slice thickness = 2 mm) covering the whole brain, FOV = 256 mm, matrix size = 128 × 128, voxel size = 2 × 2 × 2 mm^3^ (matrix size = 256 × 256, 1 × 1 × 1 mm^3^). The CT data were acquired according to scanning parameters (120 kv, 50 mA, 4.2 s, FOV: 600 mm, matrix: 512 × 512, pitch: 0.671, rotation time: 0.5 s). To achieve a spatial resolution consistent with the previous PET scanner, the PET images were reconstructed using ordered subset expectation maximization (3 iterations, 12 subsets).

### Analysis of FMT-PET image data

FMT is a more complete representative for the extent of AADC activity compared with the conventional tracer 6-[^18^F]fluoro-l-dopa (FDOPA) because FMT is not metabolized by catechol-*O*-methyl-transferase and has approximately twice the sensitivity of FDOPA. The acquired PET images were corrected by the attenuation map from the CT transmission image. The uptake of FMT was quantified by the standardized uptake value (SUV), which was calculated as follows:

$$\mathrm{SUV}=\frac{\text{Radioactive}\;\text{concentration}\;\text{(MBq/g)}}{\text{Injected}\;\text{dose}\;\text{of}\;\text{FMT}\;\text{MBq/Patient}\;\text{body}\;\text{weight}\;\text{(g)}}$$

Coordination of baseline and post-treatment PET data was then realigned. Subsequently, each PET image was co-registered into the T1-weighted MR image, using SPM 12 (Welcome Department of Cognitive Neurology, London, UK). To normalize the FMT uptake across image acquisition time points, the occipital region based on the Harvard-Oxford atlas^8^ was selected as the baseline. Applying inverse affine transformation, the template of the occipital region was registered to each PET image to extract the mean FMT uptake of the occipital lobe. Subsequently, the voxel values of PET images were converted into a ratio of the occipital region (region-to-occipital ratio) following previously reported methods^1^ as follows:

$$\text{Region}-\text{to}-\text{occipital}\;\text{ratio}=\frac{\text{FMT}\;\text{uptake}\;\text{of}\;\text{the}\;\text{target}\;\text{region}}{\text{Mean}\;\text{FMT}\;\text{uptake}\;\text{of}\;\text{the}\;\text{occipital}\;\text{lobe}}$$

Next, the FMT uptakes in the subcortical regions were extracted from the region of interest (ROI) analysis. The subcortical structures (putamen, caudate nucleus, globus pallidus and thalamus) were segmented separately as mask images from the individual T1-weighted MR image of each patient using the FSL/FIRST subcortical segmentation tool.^9^ The globus pallidus masks were divided into the globus pallidus externus and internus by a probabilistic atlas.^10^ The substantia nigra, the subthalamic nucleus, the ventral tegmental area and the cerebellar hemisphere were also extracted using the probabilistic atlas.^11–13^ The deep cerebellar nuclei were manually extracted based on the hypointensity region of the cerebellar white matter from individual diffusion tensor images. All cortical and subcortical masks were registered to the native space of each patient, applying the inverse affine transformation.

To compare FMT-PET images between at baseline, 6M post-treatment and 2Y post-treatment, as preprocessing, the individual FMT-PET images were normalized by adopting the affine transformation matrix from the T1-weighted MR image to the paediatric brain template^14^ and smoothed with a 3-mm full-width at half-maximum Gaussian kernel. Voxel-wise analysis of covariance (ANCOVA) was performed with adjustment for the age at the time of gene therapy. The contrast was estimated using a pairwise *t*-test (uncorrected *P *<* *0.001) with the spatial extent threshold determined by family-wise error correction at cluster size at *P *<* *0.05.

### 3D model reconstruction

A 3D model of the bilateral putamen was constructed from FMT-PET and MRI images using the PMOD 2.6 software package (PMOD Technologies Ltd, Zurich, Switzerland). The locations of the vector infusion in the putamen were verified by visually inspecting the four needle track marks on the postoperative T2-weighted MR image. The putaminal areas were segmented based on the maximum and 95th percentile of FMT uptake value at baseline.

### Tract analysis and connectivity-based parcellation

Probabilistic tractography at both baseline and 2Y post-treatment was performed to obtain cortico-putaminal connection areas within the putamen. All processes were performed using the FMRIB software library (FSL: version 5.0.9) and the FMRIB’s diffusion toolbox.^15^^,^^16^ Source data were corrected for eddy currents^17^ and head motion by registering all data to the first *b* = 0 image with affine transformation. The voxel-wise principal diffusion direction was estimated using FSL’s BEDPOSTX, with two fibres modelled per voxel and 5000 iterations.^18^ Subsequently, FSL’s PROBTRAX was used to determine probabilistic tractography to model 5000 iterations per voxel, with a 0.2 curvature threshold, using the single putamen seed ROI and target ROIs.

To calculate cortico-putaminal connection areas, we performed the structural connectivity-based parcellation. We adopted the 1000 cortical ROIs belonging to the seven cortical networks (visual network, somato/motor network, dorsal attention network, ventral attention network, limbic network, frontoparietal control network and default network) based on the 1489 resting-state functional magnetic resonance imaging (fMRI) scans.^19^ All ROIs were registered to the native space of each patient in DTI, applying the inverse affine transformation. All tracts were calculated in native diffusion space. The output images of cortico-putaminal connection areas were acquired by applying the ‘find_the_biggest’ FSL function. This function classifies each putaminal voxel using label numbers, corresponding to each cortical ROI, on the basis of the most significant number of paths across the ROIs. The volume of the cortico-putaminal connection area connected to each of the 1000 cortical ROIs was calculated as follows:

Volume of cortico-putaminal connection area of a cortical ROI (%)

$$=\frac{\text{All}\;\text{voxels}\;\text{of}\;\text{putaminal}\;\text{areas}\;\text{connected}\;\text{to}\;a\;\text{cortical}\;\text{ROI}}{\text{Total}\;\text{voxels}\;\text{of}\;\text{the}\;\text{whole}\;\text{putamen}}\times 100$$

The volumes of the cortico-putaminal connection areas of the 1000 cortical ROIs were summed up based on the classification of the cortical ROIs into the seven cortical networks and structural regions [PFC, PMC, SMA and primary motor cortex (M1)]. To define the ROIs of the PFC, PMC, SMA and M1, we adopted the human motor area template^20^ and the centroid coordinates of the parcellation area for the 1000 cortical ROIs. The SMA ROIs included the pre-SMA area. The parts of ROIs on the boundary area between the PMC, SMA and PFC were assigned to the PFC. All ROIs were registered to the native space of each patient in DTI. The similarity in the proportion of the whole cortico-putaminal connection area of the seven cortical networks was calculated as the paired data of each patient using the cosine similarity (0: no match; 1: the same pattern) and then averaged across patients.

To acquire the highly transduced cortico-putaminal connection area, the structural connectivity-based parcellation areas at baseline and at 2Y post-treatment were masked by the putaminal area that exceeded the maximum baseline value defined by FMT-PET images at 6M and 2Y post-treatment, respectively. The FMT-PET images were resliced into the individual DTI native space beforehand. The volume of the highly transduced cortico-putaminal connection areas connecting to each of the 1000 cortical ROIs was calculated as follows:

Volume of highly transduced cortico-putaminal connection area of a cortical ROI (%)

$$=\frac{\text{All}\;\text{voxels}\;\text{of}\;\text{highly}\;\text{transduced}\;\text{putaminal}\;\text{areas}\;\mathrm{connected}\;\text{to}\;\text{a}\;\text{cortical}\;\text{ROI}}{\text{Total}\;\text{voxels}\;\text{of}\;\text{the}\;\text{whole}\;\text{putamen}}\times 100$$

The volume of a highly transduced cortico-putaminal connection area connecting to each of the 1000 cortical ROIs was summed up based on the classification of the cortical ROIs into the seven cortical networks and structural regions (PFC, PMC, SMA and M1). In addition, the total volume of the highly transduced prefrontal cortico-putaminal connection areas in the frontoparietal control network was calculated by summing up the highly transduced putaminal area connecting to both the PFC and the frontoparietal control network.

To visualize the area of the 1000 cortical ROIs that connects to the whole and highly transduced putaminal areas across patients, we adopted the connectome workbench visualization software^21^ and the parcellation map of the 1000 cortical ROIs.^19^ Individual cortical maps of patients are combined to define the cortical areas connecting to the whole and highly transduced putaminal areas across patients. In addition, for visualization purposes, the highly transduced putaminal areas connecting to the PFC, PMC, SMA, M1 and PFC in the frontoparietal control network were normalized with the MNI standard brain template (the Montreal Neurological Institute). Then we visualized the overlapped areas in all normalized images of two or more patients.

### Evaluation of motor function

Alberta infant motor scale (AIMS) was used to observe and evaluate the patients’ gross motor development.^22^ The motor skills in four positions (prone, supine, sitting and standing) were evaluated using the following three criteria: Weight-bearing, posture, and antigravity movements. To evaluate the acquisition of new motor skills after the gene therapy, we also adopted the raw Cognition–Adaptation (C–A) score from the Kyoto Scale of Psychological Development 2001.^23^ Data from Pt. 5 and 7 were not acquired because of the unavailability of the translated version of this cognitive test. These clinical evaluations were performed by specially trained psychotherapists. Some AIMS and C-S scores (Pt. 1–6) have been described in our previous study.^2^

### Statistical analysis

A paired two-tailed *t*-test was conducted to compare the FMT uptake between at baseline and at 6M and 2Y post-treatment. Partial correlations of Spearman’s rank correlation considering the patient age at the time of gene therapy were calculated to determine the relationship of AIMS with the volume of the highly transduced cortico-putaminal connection area. To determine whether there was a significant volume of the highly transduced cortico-putaminal connection area of structural regions (PFC, PMC, SMA and M1) and seven cortical networks across patients, a two-tailed one-sample *t-*test was performed to test whether mean volumes (%) of the highly transduced cortico-putaminal connection area of structural regions (Supplementary Table 2) and seven cortical networks (Supplementary Table 3) exceeded the hypothesized mean of 0%. In addition, to examine whether the prefrontal cortico-putaminal connection area of the frontoparietal control network occupied the entire highly transduced prefrontal cortico-putaminal connection area, we performed a two-tailed paired sample *t*-test to compare the highly transduced prefrontal cortico-putaminal connection area (%) of the frontoparietal control network with that of each of the other three cortical networks (ventral attention network, limbic network and default network) (Supplementary Table 4). The *P* value was adjusted using the Holm–Bonferroni correction in case of multiple comparisons. All statistical analyses were performed using MATLAB 2018b (The MathWorks, Inc., Natick, MA, USA).

### Data availability

The data and analysis code are available from the corresponding author upon reasonable request.

## Results

### Demographic features and therapeutic effects on motor function

We examined eight patients with various types of AADC deficiency (five males and three females, age range 4–19 years, Supplementary Table 1) who received the AADC gene therapy to the bilateral putamen in an open-label phase 1/2 study (Fig. 1B; Supplementary Fig. 1). After the gene therapy, all patients showed the disappearance of painful dystonia.^2^ To evaluate motor improvement, we adopted the AIMS, which assesses gross motor development in terms of the ability to integrate antigravity muscle control in the supine, dorsal, sitting, and standing postures.^22^ A continuous gross motor improvement scored by AIMS was observed from the baseline to 6M and 2Y post-treatment [baseline vs. 6M: *t*_(7)_ = 7.05, confidence interval (CI) = 2.73–14.52, *P *=* *0.021; baseline vs. 2Y: *t*_(6)_ = 4.32, CI = 6.14–22.14, *P *=* *0.015; 6M vs. 2Y: *t*_(6)_ = 3.39, CI = 2.07–12.79, *P *=* *0.018; Fig. 1C]. In addition to AIMS, the Cognition–Adaptation score from the Kyoto Scale of Psychological Development showed the acquisition of new motor skills after gene therapy, including voluntary arm extension, grabbing, and holding objects (Fig. 1D). The behavioural results (Fig. 1C and D) confirmed the improvement of motor functions after gene therapy irrespective of patient age at the time of treatment and despite the heterogeneous genetic background of patients.

### Spatial extent of long-term transduction efficiency in gene therapy

**Figure 2:**
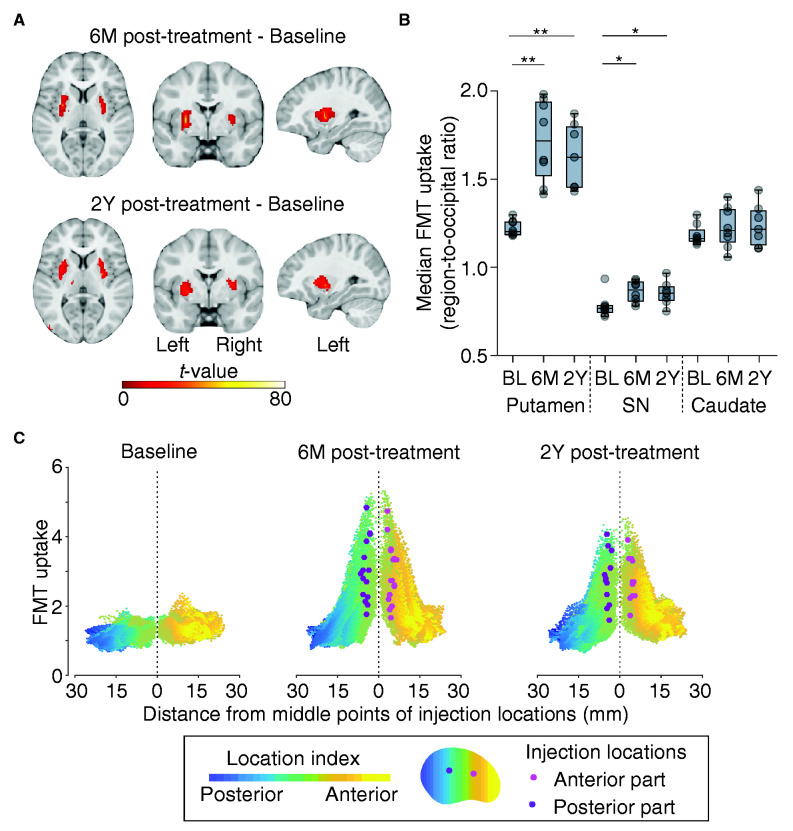
FMT-PET images of gene therapy for AADC deficiency. (**A)** Clusters of increased FMT uptake observed persistently only in the putamen at 6M and 2Y post-treatment (uncorrected *P *<* *0.001, FWE correction at cluster level *P *<* *0.05). **(B)** Median FMT uptake in the putamen, substantia nigra (SN), and caudate. Significant increases were found in the putamen and SN after gene therapy (see Supplementary Fig. 4 for details). *P <* *0.05, **P <* *0.01. **(C)** Spatial distribution of FMT uptake. It revealed decreased FMT uptake from vector injection locations. The mean maximum distant point that exceeded the maximum FMT uptake at baseline was 11.56 ± 1.40 mm from the injection point at 6M post-treatment and 10.92 ± 1.43 mm at 2Y post-treatment. The location index reflects the coordination in the putamen.

To evaluate the transduced putaminal area that receives cortical projections at a high spatial resolution, we examined the spatial and temporal aspects of the transduction efficiency using high spatial resolution PET with the radiotracer 6-[^18^F]fluoro-l-*m*-tyrosine (FMT). FMT-PET images were acquired at baseline (pretreatment), 6M (3.5 months for Pt. 5), and 2Y post-treatment. The uptake of FMT in the putamen was minimal at baseline, significantly increased at 6M post-treatment, and has been sustained for 2 years in all patients (putamen: 6M, *t*_(7)_ = 6.41, CI = 0.31–0.68, *P *=* *0.0012; 2Y, *t*_(6)_ = 6.50, CI = 0.25–0.56, *P *=* *0.0012; Fig. 2A; Supplementary Figs 2 and 3). No robust increase in FMT uptake for 2 years was observed in the cortical regions. In addition to the putamen, there were significant increases in the median FMT uptake in the globus pallidus and substantia nigra (Fig. 2B; Supplementary Fig. 4A), indicating possible anterograde transportation given that AAV2 has not been reported to retrogradely transport.^24^^,^^25^ Moreover, deep brain regions, including the ventral tegmental area and deep cerebellar nuclei that modulate dopaminergic signalling, exhibited no increased FMT uptake (Supplementary Fig. 4B). The spatial distribution of FMT uptake in the putamen and the 3D models demonstrated that the increased uptake area was broadly distributed from the locations of the vector-delivered needle tracks to the anterior and dorsal parts of the putamen (Fig. 2C; Supplementary Fig. 5). The highly transduced areas over the maximum uptake value at baseline constituted 44.75% ± 5.60% (mean ± SEM) and 38.47% ± 6.42% at 6M and 2Y post-treatment, respectively, of the entire putamen (6M vs. 2Y, *t*_(6)_ = 1.48, CI = −2.42 to 9.81, *P *=* *0.11). Furthermore, the transduced areas over the 95th percentile uptake value at baseline comprised 64.5% ± 5.38% and 61.12% ± 5.29% at 6M and 2Y post-treatment, respectively (6M vs. 2Y, *t*_(6)_ = 1.00, CI = −6.44 to 9.35, *P *=* *0.67). The volume of the highly transduced area was sustained for 2 years and was not associated with the distance between two injection points (6M post-treatment: *ρ* = 0.47, *P *=* *0.066; 2Y post-treatment: *ρ* = 0.46, *P *=* *0.15). Our results suggest that a significant transduction occurs only in the putamen and that the highly transduced putaminal area has been sustained at the centre of the vector injection locations for 2 years. Indeed, follow-up tests focused on catecholamine of the cerebrospinal fluid in our previous study^2^ showed an increase in HVA and a reduction in levodopa (l-dopa) after gene therapy, thus indicating the sustained expression of functional AADC.

### Cortico-putaminal connection in the highly transduced putaminal area

**Figure 3:**
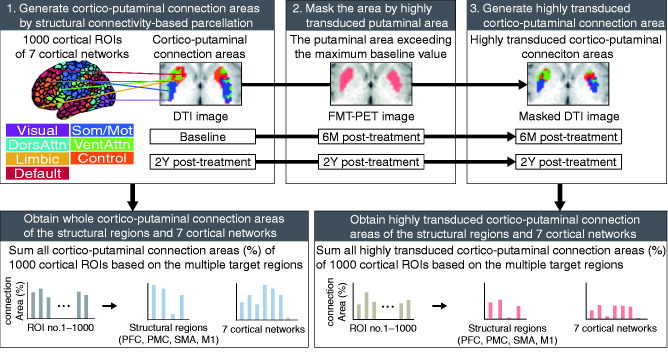
**Schematic diagram to extract highly transduced cortico-putaminal connection areas from the 1000 cortical ROIs of seven cortical networks.** Using the structural connectivity-based parcellation to the DTI image at baseline and 2Y post-treatment, the cortico-putaminal connection areas were calculated between the putaminal area and 1000 cortical ROIs of the seven cortical networks. The cortico-putaminal connection areas were combined based on the classification of the 1000 cortical ROIs into the structural regions (PFC, PMC, SMA and M1) and seven cortical networks. To produce highly transduced cortico-putaminal connection areas, the whole cortico-putaminal connection areas at baseline and 2Y post-treatment were masked by the highly transduced area of the FMT-PET images at 6M and 2Y post-treatment, respectively. The highly transduced area was defined as the area that exceeds the maximum FMT uptake at baseline. We calculated the highly transduced connection areas (%) of 1000 cortical ROIs and then combined them based on their structural regions and seven cortical networks.

**Figure 4:**
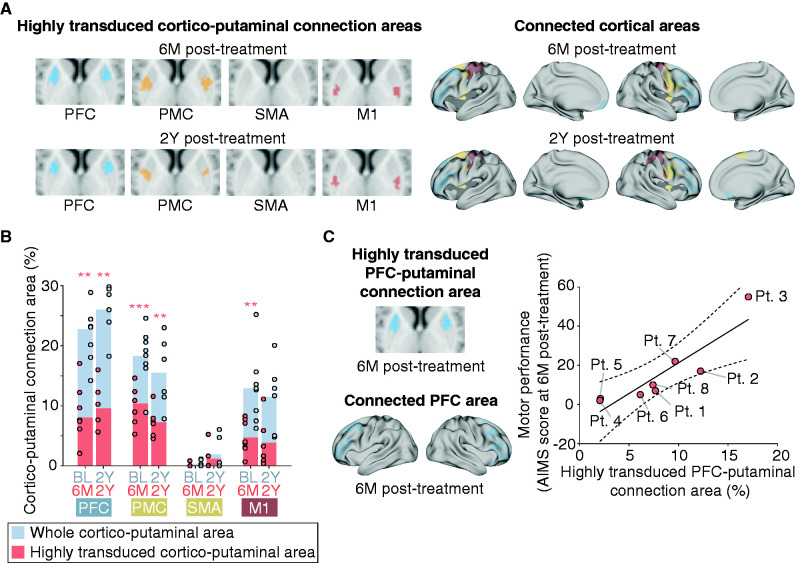
**Connection areas between structural regions of motor system and highly transduced putamen and its association with gross motor improvement.** (**A)** Highly transduced cortico-putaminal connection areas and connected cortical areas in the motor system across patients. Black-coloured areas in the cortical model represent the cortical areas except PFC, PMC, SMA and M1 that connected to the highly transduced putaminal area. (**B)** Highly transduced cortico-putaminal connection area in the motor system. Gray-coloured bars represent the whole cortico-putaminal connection area at baseline and 2Y post-treatment. Pink-coloured bars represent the highly transduced cortico-putaminal connection area at 6M and 2Y post-treatment (see Fig. 3 for details). Pink-coloured asterisks represent the significant volumes of the highly transduced cortico-putaminal connection area. **P <* *0.01, ***P <* *0.001. (**C)** Relationship between the highly transduced prefrontal cortico-putaminal connection area and motor performance at 6M post-treatment. Putaminal and cortical images represent the highly transduced cortico-putaminal connection areas and cortical areas connecting to the putaminal area across patients, respectively. The raw data are plotted in the right panel. For the partial correlation analysis, the age of the treatment was adopted. Dashed line: 95% confidence interval.

The persistent expression of AADC in the specific putaminal area over the years indicates the constant dopaminergic restoration in the distinct cortico-putaminal network after the AADC gene therapy, which resulted in the continuous motor improvement for 2 years. Therefore, we examined the topographic organization of the highly transduced putaminal areas based on structural connections to cortical areas. Using the structural connectivity-based parcellation between the putamen and cortical areas to diffusion tensor images at baseline and 2Y post-treatment, we obtained putaminal parcellation maps defined as cortico-putaminal connection areas at baseline and 2Y post-treatment (Fig. 3). To generate the fine cortico-putaminal connection areas of both motor system and striatum-related cortical networks, we adopted 1000 cortical regions of interest (ROIs) belonging to the seven cortical networks that were extracted from resting-state fMRI data of 1489 participants^19^ and were also associated with striatal functional properties.^3–5^ To obtain the highly transduced cortico-putaminal connection areas, the cortico-putaminal connection areas were segmented on the basis of the highly transduced putaminal areas that exceeded the maximum baseline value. To examine the cortico-putaminal networks that were estimated to recover functionally by dopamine restoration at 6M post-treatment, the cortico-putaminal connection areas at baseline were segmented with the highly transduced putaminal areas at 6M post-treatment. To examine sustainably functioning cortico-putaminal networks by persistent dopamine restoration for 2 years, the cortico-putaminal connection areas at 2Y post-treatment were segmented with the highly transduced putaminal areas at 2Y post-treatment. First, we investigated the possible association between the motor performance and the highly transduced putaminal areas connecting to cortical areas in the motor system (PFC, PMC, SMA and M1) (Fig. 4A). The highly transduced putaminal area across patients was connected to the PFC, PMC and M1 at 6M post-treatment and PFC and PMC at 2Y post-treatment (Fig. 4B), indicating that PFC and PMC were primarily connected to the highly transduced putaminal area across patients for 2 years. The partial correlation adjusted for age of the treatment showed that the highly transduced putaminal area connecting to PFC (prefrontal cortico-putaminal connection area) was significantly associated with motor performance at 6M post-treatment (Spearman’s partial correlation coefficient adjusted for patient age at the time of treatment: *ρ* = 0.97, *P *=* *0.0014; Fig. 4C), whereas no correlation was found regarding other cortical regions (SMA, no highly transduced cortico-putaminal connection area in all patients except Pt. 3; PMC, *ρ* = 0.12, *P *=* *0.80; M1, *ρ* = 0.52, *P *=* *0.23).

**Figure 5:**
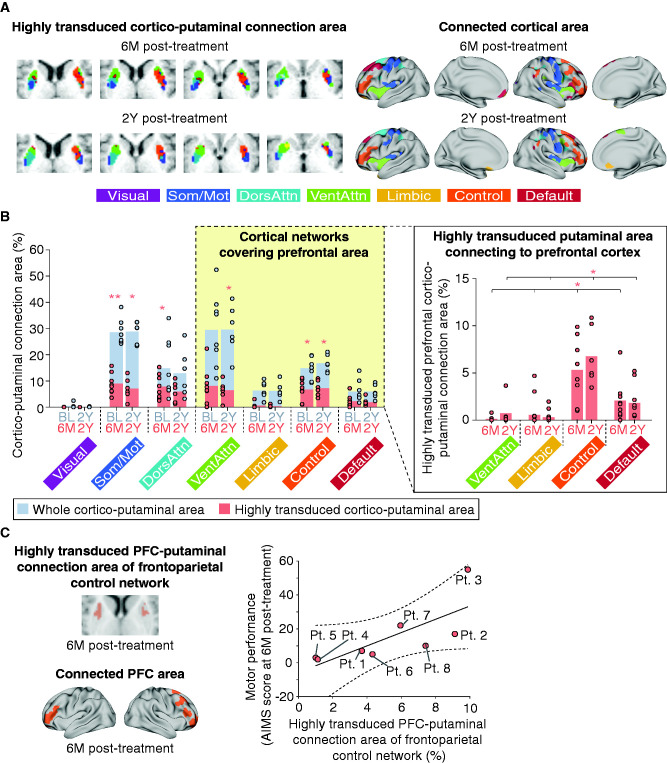
**Connection areas between cortical networks and highly transduced putamen and its association with gross motor improvement.** (**A)** Representative examples of highly transduced cortico-putaminal connection areas from Pt. 1 and cortical areas connecting to the highly transduced putaminal area across patients (see Supplementary Figs 6 and 7 for details). Som/Mot: somato/motor network; DorsAttn: dorsal attention network; VentAttn: ventral attention network; Control: frontoparietal control network. **(B)** The whole cortico-putaminal connection area (left panel) and highly transduced prefrontal cortico-putaminal connection area (right panel) of seven cortical networks. Gray-coloured bars represent the whole cortico-putaminal connection area at baseline and 2Y post-treatment. Pink-coloured bars represent the highly transduced cortico-putaminal connection area at 6M and 2Y post-treatment (see Fig.3 for details). Pink asterisks in the left and right panels represent significant volumes of highly transduced cortico-putaminal connection area (Supplementary Table 3) and significant difference between the prefrontal cortico-putaminal connection area of the frontoparietal control network and that of the other cortical networks (Supplementary Table 4). *P <* *0.05, **P <* *0.01. **(C)** Relationship between the prefrontal cortico-putaminal connection area of the frontoparietal control network and the motor performance at 6M post-treatment. The raw data are plotted in the right panel. Partial correlation analysis was performed after adjustment for patient age at the time of treatment.

Next, to evaluate the major cortical network in the highly transduced prefrontal cortico-putaminal connection areas, we examine the cortico-putaminal connection areas of the seven striatum-related cortical networks^3–5^ (Fig. 5A). The spatial organization of the connection areas at baseline was found to be similar to one observed in functional connectivity studies of healthy human children and adults^5^^,^^26^ and sustained for 2 years (Supplementary Fig. 6). The proportion of the cortico-putaminal connection areas of the seven cortical networks was coherent across patients [baseline: 0.86 ± 0.021 (mean cosine similarity ± SEM), 2Y post-treatment: 0.91 ± 0.019]. The cortical networks that were connected to the significant volumes of the highly transduced putaminal areas across patients were the frontoparietal control network, the somato/motor network, and the dorsal attention network at 6M post-treatment. We also found the significant volumes of the highly transduced putaminal areas across patients were connected to the frontoparietal control network, the somato/motor network and the ventral attention network at 2Y post-treatment. The cortical areas that connected to the highly transduced putaminal area covered the PFC, especially the dorsolateral prefrontal cortex, the ventrolateral prefrontal cortex and the orbitofrontal cortex in the frontoparietal control network, the PMC and M1 in the somato/motor network, the frontal eye field in the dorsal attention network, and the insular cortex in the ventral attention network, respectively (Supplementary Fig. 7). The result shows that the frontoparietal control network and the somato/motor network robustly connected to the highly transduced putaminal areas across patients for 2 years (Fig. 5B, left panel; Supplementary Table 3). Moreover, the frontoparietal control network was found to be the dominant network of the highly transduced prefrontal cortico-putaminal connection area (Fig. 5B, right panel; Supplementary Table 4). The highly transduced prefrontal cortico-putaminal connection area of the frontoparietal control network also exhibited an association with the motor performance at 6M post-treatment (Spearman’s partial correlation coefficient adjusted for patient age at the time of treatment: *ρ* = 0.89, *P *=* *0.0076; Fig. 5C). These results suggest that the cortico-putaminal network was established to some extent, even under dopamine deficiency, and also suggest that the motor system recovery is attributed to the dopamine replacement in the existing cortico-putaminal network, especially the prefrontal cortico-putaminal network in the frontoparietal control network, by the AADC gene therapy. Therefore, the dopaminergic restoration of putaminal connection to the PFC for motor intention contributes to the gross motor improvement after the AADC gene therapy.

## Discussion

Our results demonstrated that the persistent dopaminergic restoration in the prefrontal cortico-putaminal network is a basis for the therapeutic effects of the AADC gene therapy on the motor system. It provides a novel insight into the mechanism underlying the motor improvement by the putaminal AADC gene therapy (Supplementary Fig. 8). From the perspective of the putaminal AADC gene therapy for Parkinson’s disease wherein the degeneration of the nigrostriatal dopaminergic pathway in the cortico-basal ganglia network leads to the development of motor symptoms, the central mechanism underlying the motor functional recovery by the putaminal AADC gene transfer has been assumed to be the restoration of the motor cortico-putaminal network to recover motor executive function.^27^ Indeed, we found the existence of a highly transduced area connected to M1 and somato/motor network (Figs 4B and 5B), which reflects the transient choreic dyskinesias observed in our patients after gene therapy.^2^ However, only dopaminergic recovery of the primary motor cortico-putaminal network cannot account for the amelioration of dystonia, cognitive improvement and acquisition of new motor skills as well as gross motor improvements observed after the gene therapy. In contrast, the PFC, frontoparietal control network and prefrontal cortico-striatal network are known to be responsible for the motor and cognitive control.^28–30^ The function of the orbitofrontal cortex, dorsolateral prefrontal cortex and ventrolateral prefrontal cortex in the motor system is the motor intention, including the selection of movements, planning of sequential movements and inhibitory control of motor responses.^31^ In fact, impaired function and structure of the frontal cortex has been reported in case of AADC deficiency as decreased FDG uptake in a PET study^32^ and less fibre development in a DTI study,^33^ which indicates that the motor control function is primarily impaired by AADC deficiency. Moreover, a recent study showed the dopamine signal in human putamen fluctuates with the time to a decision-making choice,^34^ which supports that the putaminal dopamine reflects motor-related activity, possibly motor intention. Together, the motor improvement associated with the gene therapy for AADC deficiency can be attributed to the dopamine-driven development and optimization of an immature top-down motor control system,^35^ possibly an internal model for motor control,^36^ in young patients with AADC deficiency rather than to the functional recovery of a well-developed motor system observed in elderly patients with Parkinson’s disease. It indicates the possible different neural mechanisms of the therapeutic effects by the putaminal AADC gene therapy between AADC deficiency and Parkinson’s disease.

Although we did not perform the longitudinal kinesiological evaluation of dystonic symptoms, it is also noteworthy that the dopaminergic intervention of the specific putaminal area by delivery of AADC-expressing AAV vectors leads to the amelioration of limb and truncal dystonia, which may provide some clues on the pathophysiological basis of dystonia. Dystonia has been related to metabolic, anatomical or functional abnormalities of the nigrostriatal pathway.^37^ Indeed, most agents related to drug-induced dystonia, including oculogyric crisis, are known to disrupt dopaminergic neurotransmission,^37^^,^^38^ implicating that dopaminergic deficit in the putamen may be related to the development of dystonia. In contrast, no FMT uptake was observed in the cerebellum and ventral tegmental area (Supplementary Fig. 4B), although the fastigial nucleus, one of the deep cerebellar nuclei, projects to the ventral tegmental area may modulate dopaminergic signalling.^39^ It suggests that this pathway is not involved in dystonia observed in AADC deficiency, supporting the notion that the cerebellum is not the primary cause of dystonia.^40^ Moreover, dystonia is considered as a network disorder comprising the basal ganglia, cerebellum and multiple cortical regions, including the PFC, PMC and primary motor cortex.^40–42^ Resting-state fMRI studies also showed reduced functional connectivity of the sensorimotor and frontoparietal control networks in patients with dystonia.^43^ These evidences are consistent with our findings that the highly transduced putaminal area predominantly connected to not only the PFC, PMC and M1 (Fig. 4B) but also somato/motor network and frontoparietal control network, which reflect the dopaminergic restoration of these networks across patients (Fig. 5B, left panel). Altogether, our results provide evidence that dystonia is a network disorder and that the dopaminergic recovery of the cortico-putaminal network might be associated with the amelioration of dystonia.

Several limitations should be mentioned in the present study. First, the study included a limited number of patients with a heterogeneous genetic background. Therefore, it remains inconclusive whether certain unknown factors, such as initial levels of dopamine and serotonin before the treatment, might have contributed to the individual differences in response to treatment. Second, while we showed that the prefrontal cortico-putaminal connection area was associated with gross motor improvement, we could not clarify specific functions of the PFC that were facilitated by the gene therapy. Most patients with AADC deficiency were in a severe clinical condition that causes immobility, developmental delay and communication disability. Therefore, it is significantly challenging to evaluate the function and development of the PFC by general cognitive and motor tests such as go/no-go and working memory tests. Finally, although FMT-PET images enable visualizing AADC activity at high spatial resolutions, the transduction extent remains controversial, given the lack of direct evidence linking FMT-PET images with human histological data. To avoid the analysis of pseudo-transduced putaminal areas, we focused on areas that exceeded the baseline FMT uptake value. However, we suppose that this is not the only transduced area because approximately 60% of the putaminal area exceeds the 95th percentile baseline value (Supplementary Fig. 5). In addition, the FMT uptake value was significantly increased, consistent with the shape of the putamen (Supplementary Figs 2 and 5) without any increase in background FMT signals. Further studies should be performed to investigate the relationship between the distribution of FMT signal intensity and actual transduction areas, the possible selective transduction of medium spiny neurons in the striosomes and matrix compartments, and the distribution of transduced D1 and D2 receptors.^24^^,^^44^

## Supplementary material

Supplementary material is available at *Brain Communications* online.

## Supplementary Material

fcab078_Supplementary_Data

## Abbreviations

AADC =: aromatic l-amino acid decarboxylase
AAV =: adeno-associated virus
AIMS =: Alberta infant motor scale
CI =: confidence interval
DDC =: dopa decarboxylase
DTI =: diffusion tensor imaging
FOV =: field of view
M1 =: primary motor cortex
PFC =: prefrontal cortex
PMC =: premotor cortex
ROI =: region of interest
SMA =: supplementary motor area
6M =: 6 months
2Y =: 2 years

## References

[1] Hwu W-L, Muramatsu S, Tseng S-H, et al Gene therapy for aromatic l-amino acid decarboxylase deficiency. Sci Transl Med. 2012;4(134):134ra61. 10.1126/scitranslmed.3003640 22593174

[2] Kojima K, Nakajima T, Taga N, et al Gene therapy improves motor and mental function of aromatic l-amino acid decarboxylase deficiency. Brain. 2019;142(2):322–333. 30689738 10.1093/brain/awy331 PMC6377184

[3] Roffman JL, Tanner AS, Eryilmaz H, et al Dopamine D 1 signaling organizes network dynamics underlying working memory. Sci Adv. 2016;2(6):e1501672. 27386561 10.1126/sciadv.1501672 PMC4928887

[4] Anderson KM, Krienen FM, Choi EY, Reinen JM, Yeo BTT, Holmes AJ. Gene expression links functional networks across cortex and striatum. Nat Commun. 2018;9(1):1428. 29651138 10.1038/s41467-018-03811-x PMC5897339

[5] Choi EY, Thomas Yeo BT, Buckner RL. The organization of the human striatum estimated by intrinsic functional connectivity. J Neurophysiol. 2012;108(8):2242–2263. 22832566 10.1152/jn.00270.2012 PMC3545026

[6] Jahanshahi M, Obeso I, Rothwell JC, Obeso JA. A fronto–striato–subthalamic–pallidal network for goal-directed and habitual inhibition. Nat Rev Neurosci. 2015;16(12):719–732. 26530468 10.1038/nrn4038

[7] Anderson ND, Dell GS. The role of consolidation in learning context-dependent phonotactic patterns in speech and digital sequence production. Proc Natl Acad Sci. 2018;115(14):3617–3622. 29555766 10.1073/pnas.1721107115 PMC5889662

[8] Desikan RS, Ségonne F, Fischl B, et al An automated labeling system for subdividing the human cerebral cortex on MRI scans into gyral based regions of interest. Neuroimage. 2006;31(3):968–980. 16530430 10.1016/j.neuroimage.2006.01.021

[9] Patenaude B, Smith SM, Kennedy DN, Jenkinson M. A Bayesian model of shape and appearance for subcortical brain segmentation. Neuroimage. 2011;56(3):907–922. 21352927 10.1016/j.neuroimage.2011.02.046 PMC3417233

[10] Keuken MC, Bazin P-L, Crown L, et al Quantifying inter-individual anatomical variability in the subcortex using 7 T structural MRI. Neuroimage. 2014;94:40–46. 24650599 10.1016/j.neuroimage.2014.03.032

[11] Murty VP, Shermohammed M, Smith DV, Carter RM, Huettel SA, Adcock RA. Resting state networks distinguish human ventral tegmental area from substantia nigra. Neuroimage. 2014;100:580–589. 24979343 10.1016/j.neuroimage.2014.06.047 PMC4370842

[12] Forstmann BU, Keuken MC, Jahfari S, et al Cortico-subthalamic white matter tract strength predicts interindividual efficacy in stopping a motor response. Neuroimage. 2012;60(1):370–375. 22227131 10.1016/j.neuroimage.2011.12.044

[13] Diedrichsen J, Balsters JH, Flavell J, Cussans E, Ramnani N. A probabilistic MR atlas of the human cerebellum. Neuroimage. 2009;46(1):39–46. 19457380 10.1016/j.neuroimage.2009.01.045

[14] Fonov V, Evans AC, Botteron K, et al; Brain Development Cooperative Group. Unbiased average age-appropriate atlases for pediatric studies. Neuroimage. 2011;54(1):313–327. 20656036 10.1016/j.neuroimage.2010.07.033 PMC2962759

[15] Smith SM, Jenkinson M, Woolrich MW, et al Advances in functional and structural MR image analysis and implementation as FSL. Neuroimage. 2004;23:S208–S219. 15501092 10.1016/j.neuroimage.2004.07.051

[16] Woolrich MW, Jbabdi S, Patenaude B, et al Bayesian analysis of neuroimaging data in FSL. Neuroimage. 2009;45(1 Suppl):S173–S186. 19059349 10.1016/j.neuroimage.2008.10.055

[17] Andersson JLR, Sotiropoulos SN. An integrated approach to correction for off-resonance effects and subject movement in diffusion MR imaging. Neuroimage. 2016;125:1063–1078. 26481672 10.1016/j.neuroimage.2015.10.019 PMC4692656

[18] Traynor C, Heckemann RA, Hammers A, et al Reproducibility of thalamic segmentation based on probabilistic tractography. Neuroimage. 2010;52(1):69–85. 20398772 10.1016/j.neuroimage.2010.04.024

[19] Schaefer A, Kong R, Gordon EM, et al Local-global parcellation of the human cerebral cortex from intrinsic functional connectivity MRI. Cereb Cortex. 2018;28(9):3095–3114. 28981612 10.1093/cercor/bhx179 PMC6095216

[20] Mayka MA, Corcos DM, Leurgans SE, Vaillancourt DE. Three-dimensional locations and boundaries of motor and premotor cortices as defined by functional brain imaging: A meta-analysis. Neuroimage. 2006;31(4):1453–1474. 16571375 10.1016/j.neuroimage.2006.02.004 PMC2034289

[21] Marcus DS, Harms MP, Snyder AZ, et al; WU-Minn HCP Consortium. Human Connectome Project informatics: Quality control, database services, and data visualization. Neuroimage. 2013;80:202–219. 23707591 10.1016/j.neuroimage.2013.05.077 PMC3845379

[22] Piper MC, Pinnell LE, Darrah J, Maguire T, Byrne PJ. Construction and validation of the Alberta Infant Motor Scale (AIMS). Can J Public Health. 1992;(83 Suppl 2):S46–S50. 1468050

[23] Koyama T, Osada H, Tsujii H, Kurita H. Utility of the Kyoto Scale of Psychological Development in cognitive assessment of children with pervasive developmental disorders. Psychiatry Clin Neurosci. 2009;63(2):241–243. 19335396 10.1111/j.1440-1819.2009.01931.x

[24] Sehara Y, Fujimoto K, Ikeguchi K, et al Persistent expression of dopamine-synthesizing enzymes 15 years after gene transfer in a primate model of Parkinson’s disease. Hum Gene Ther Clin Dev. 2017;28(2):74–79. 28279081 10.1089/humc.2017.010

[25] Salegio EA, Samaranch L, Kells AP, et al Axonal transport of adeno-associated viral vectors is serotype-dependent. Gene Ther. 2013;20(3):348–352. 22418061 10.1038/gt.2012.27 PMC3381869

[26] Cignetti F, Nemmi F, Vaugoyeau M, et al Intrinsic cortico-subcortical functional connectivity in developmental dyslexia and developmental coordination disorder. Cereb Cortex Commun. 2020;1(1):tgaa011. 34296090 10.1093/texcom/tgaa011 PMC8152893

[27] Muramatsu S, Fujimoto K, Kato S, et al A phase I study of aromatic l-amino acid decarboxylase gene therapy for Parkinson’s disease. Mol Ther. 2010;18(9):1731–1735. 20606642 10.1038/mt.2010.135 PMC2956925

[28] Haggard P. Human volition: Towards a neuroscience of will. Nat Rev Neurosci. 2008;9(12):934–946. 19020512 10.1038/nrn2497

[29] Woolgar A, Hampshire A, Thompson R, Duncan J. Adaptive coding of task-relevant information in human frontoparietal cortex. J Neurosci. 2011;31(41):14592–14599. 21994375 10.1523/JNEUROSCI.2616-11.2011 PMC6703398

[30] Terra H, Bruinsma B, de Kloet SF, van der Roest M, Pattij T, Mansvelder HD. Prefrontal cortical projection neurons targeting dorsomedial striatum control behavioral inhibition. Curr Biol. 2020;30(21):4188–4200.e5. 32888489 10.1016/j.cub.2020.08.031

[31] Tanji J. Sequential organization of multiple movements: Involvement of cortical motor areas. Annu Rev Neurosci. 2001;24(1):631–651. 11520914 10.1146/annurev.neuro.24.1.631

[32] Ide S, Sasaki M, Kato M, et al Abnormal glucose metabolism in aromatic l-amino acid decarboxylase deficiency. Brain Dev. 2010;32(6):506–510. 19520530 10.1016/j.braindev.2009.05.004

[33] Lee W, Lin J, Weng W, Peng SS. Microstructural changes of brain in patients with aromatic l-amino acid decarboxylase deficiency. Hum Brain Mapp. 2017;38(3):1532–1540. 27859928 10.1002/hbm.23470 PMC6867156

[34] Bang D, Kishida KT, Lohrenz T, et al Sub-second dopamine and serotonin signaling in human striatum during perceptual decision-making. Neuron. 2020;108(5):999–1010.e6. 33049201 10.1016/j.neuron.2020.09.015 PMC7736619

[35] Narayanan NS, Laubach M. Top-down control of motor cortex ensembles by dorsomedial prefrontal cortex. Neuron. 2006;52(5):921–931. 17145511 10.1016/j.neuron.2006.10.021 PMC3995137

[36] Egger SW, Remington ED, Chang C-J, Jazayeri M. Internal models of sensorimotor integration regulate cortical dynamics. Nat Neurosci. 2019;22(11):1871–1882. 31591558 10.1038/s41593-019-0500-6 PMC6903408

[37] Barow E, Schneider SA, Bhatia KP, Ganos C. Oculogyric crises: Etiology, pathophysiology and therapeutic approaches. Parkinsonism Relat Disord. 2017;36:3–9. 27964831 10.1016/j.parkreldis.2016.11.012

[38] Ribot B, Aupy J, Vidailhet M, et al Dystonia and dopamine: From phenomenology to pathophysiology. Prog Neurobiol. 2019;182:101678. 31404592 10.1016/j.pneurobio.2019.101678

[39] Carta I, Chen CH, Schott AL, Dorizan S, Khodakhah K. Cerebellar modulation of the reward circuitry and social behavior. Science. 2019;363(6424):eaav0581. 30655412 10.1126/science.aav0581 PMC6711161

[40] Kaji R, Bhatia K, Graybiel AM. Pathogenesis of dystonia: Is it of cerebellar or basal ganglia origin? J Neurol Neurosurg Psychiatry. 2018;89(5):488–492. 29089396 10.1136/jnnp-2017-316250 PMC5909758

[41] Carbon M, Eidelberg D. Abnormal structure-function relationships in hereditary dystonia. Neuroscience. 2009;164(1):220–229. 19162138 10.1016/j.neuroscience.2008.12.041 PMC2760608

[42] Battistella G, Simonyan K. Top-down alteration of functional connectivity within the sensorimotor network in focal dystonia. Neurology. 2019;92(16):e1843–e1851. 30918091 10.1212/WNL.0000000000007317 PMC6550502

[43] Battistella G, Termsarasab P, Ramdhani RA, Fuertinger S, Simonyan K. Isolated focal dystonia as a disorder of large-scale functional networks. Cereb Cortex. 2017;27(2):1203–1215. 26679193 10.1093/cercor/bhv313 PMC6075177

[44] McGregor MM, Nelson AB. Circuit mechanisms of Parkinson’s disease. Neuron. 2019;101(6):1042–1056. 30897356 10.1016/j.neuron.2019.03.004

