# Supplementary material for: Dopaminergic restoration of prefrontal cortico-putaminal network in gene therapy for aromatic l-amino acid decarboxylase deficiency
Source: Brain Commun. 2021 Apr 15;3(3):fcab078. doi: 10.1093/braincomms/fcab078 (PMC8374966; doi:10.1093/braincomms/fcab078)
Supplement: fcab078_Supplementary_Data [file fcab078_Supplementary_Data.pdf]

Supplementary Materials

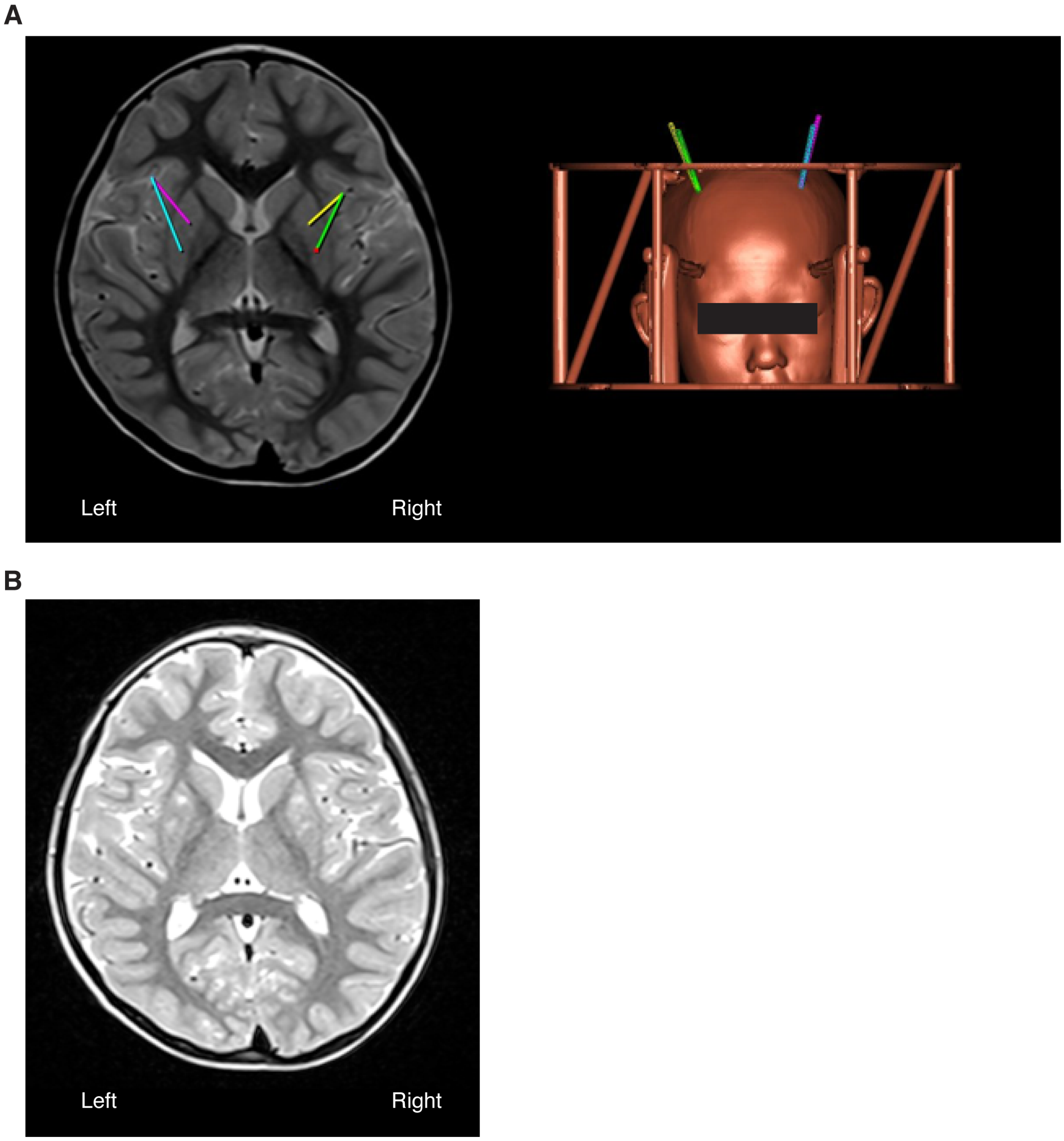

Supplementary Figure 1. Representative image of the surgical plan and postoperative T2-weighted MR image. (A) Images of the planned injection locations (purple-, blue-, yellow-, and green-colored lines) of the putamen (left images) and reconstructed image of stereotactic neurosurgery (right image). **(B)** Postoperative T2-weighted MR image. The four white needle track marks found in the image confirmed the precise injection of the AAV-h*AADC*-2 vectors into the planned injection locations of the putamen.

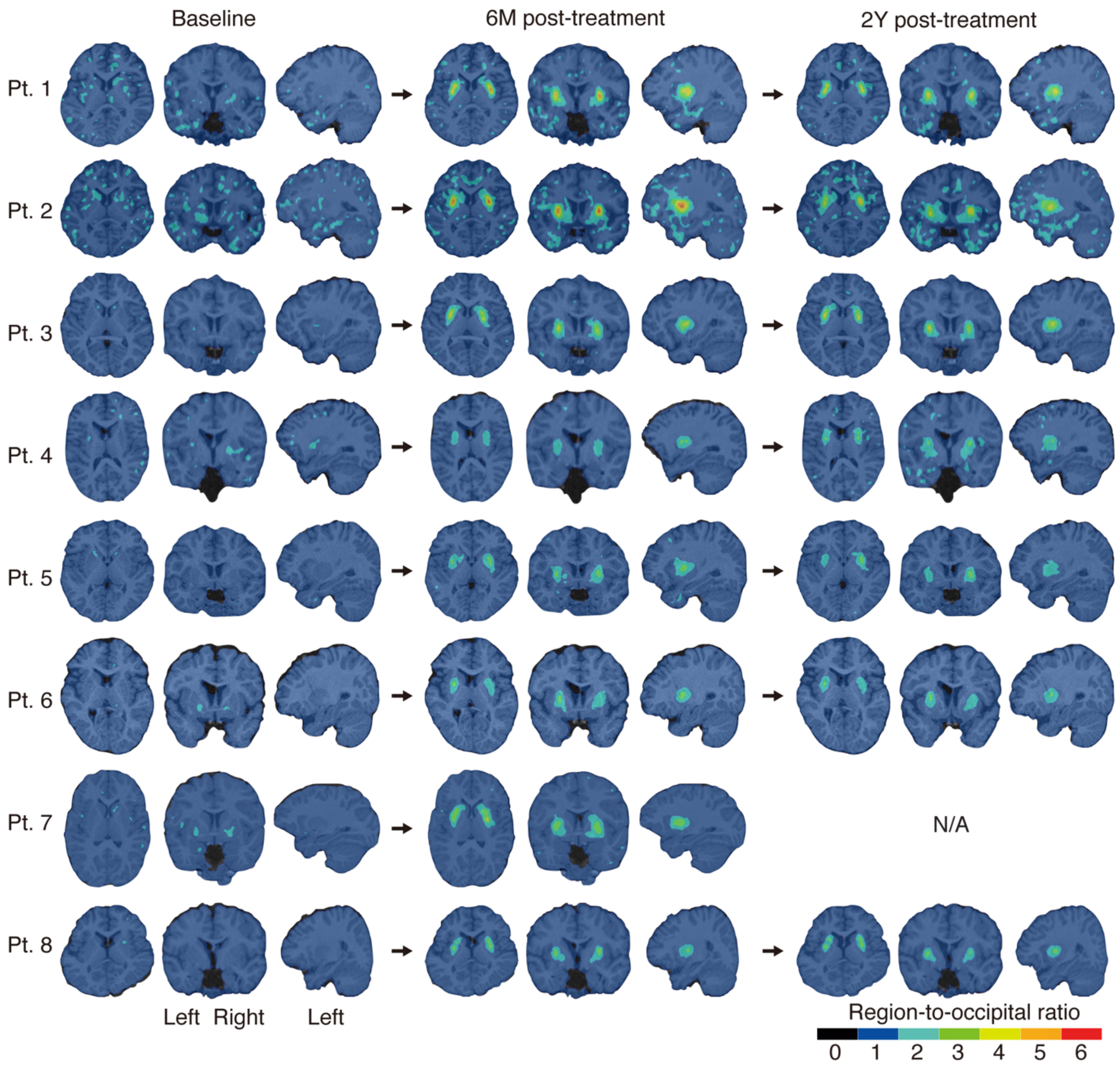

Supplementary Figure 2. FMT-PET images of AADC deficiency in patients. Individual FMT-PET images were fused with T1-weighted MR images. FMT uptake (region-to-occipital ratio) of all images is a discrete value for visualization purposes.

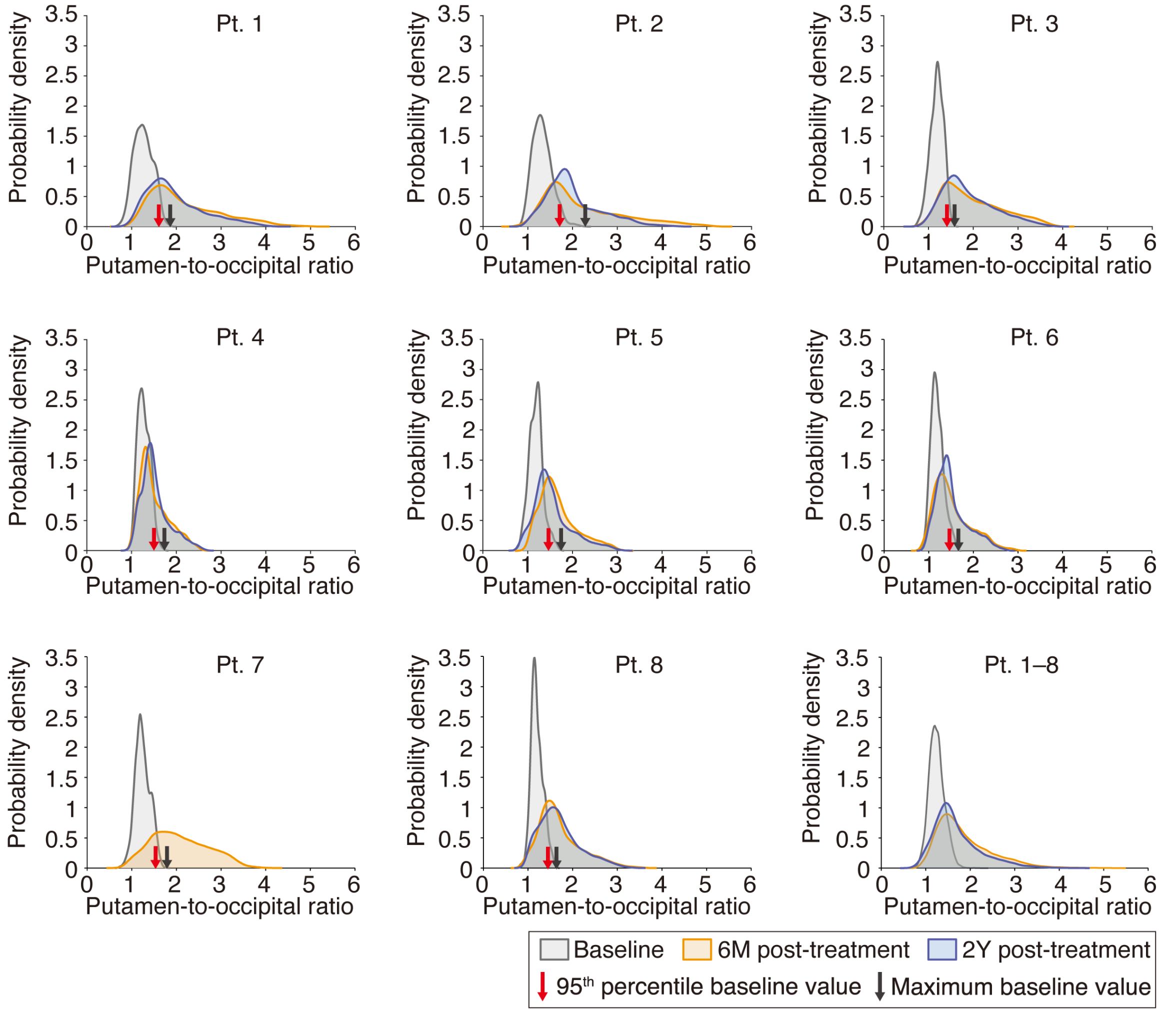

Supplementary Figure 3. FMT uptake distribution in the putamen at baseline and post-treatment. The distribution of FMT uptake in the putamen positively skewed from the baseline to 6M and 2Y post-treatment. The x and y axes denote the putamen-to-occipital ratio (FMT uptake) and the probability density, respectively. Red and black arrows denote the cutoff value obtained from the 95th percentile baseline value and the maximum baseline value of FMT uptake, respectively.

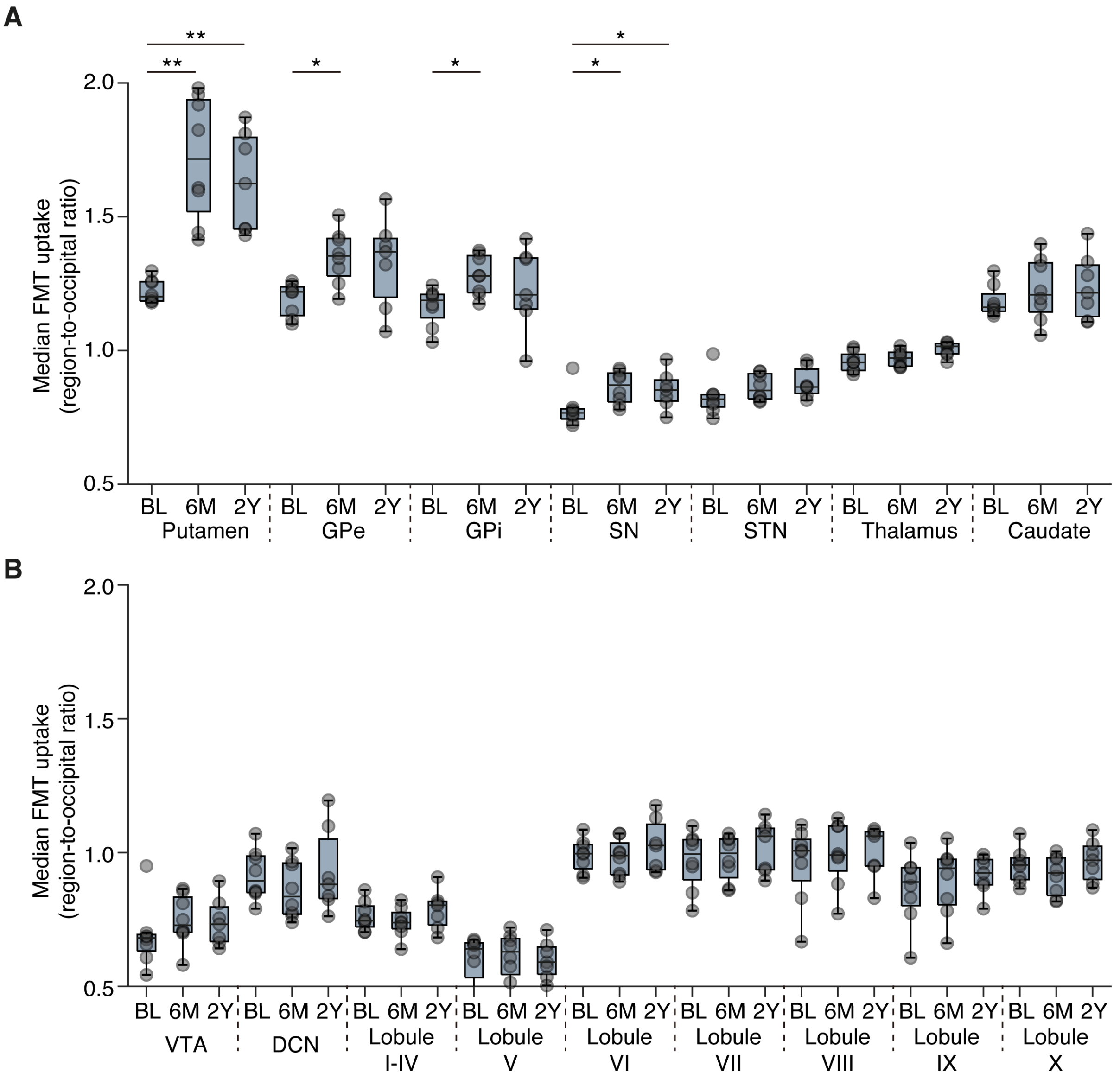

Supplementary Figure 4. FMT uptake of subcortical and cerebellar regions. (A) Median FMT uptakes in the basal ganglia. In addition to the putamen, significant increases in the median FMT uptake were found in the globus pallidus externus (GPe), globus pallidus internus (GPi), and substantia nigra (SN) after gene therapy (GPe: 6M, $t_{(7)}$ = 3.71, 95% CI = 0.058–0.26, P = 0.023; GPi: 6M, $t_{(7)}$ = 3.60, 95% CI = 0.040–0.19, P = 0.026; SN: 6M, $t_{(7)}$ = 3.11, 95% CI = 0.027–0.14, P = 0.028; 2Y, $t_{(6)}$ = 3.68, 95% CI = 0.024– 0.12, P = 0.021). *P < 0.05, **P < 0.01. STN, subthalamic nucleus. (**B)** Median FMT uptake in the VTA, DCN, and cerebellar hemisphere. No increased FMT uptake was found in these regions (P values > 0.05). VTA: ventral tegmental area; DCN: deep cerebellar nuclei.

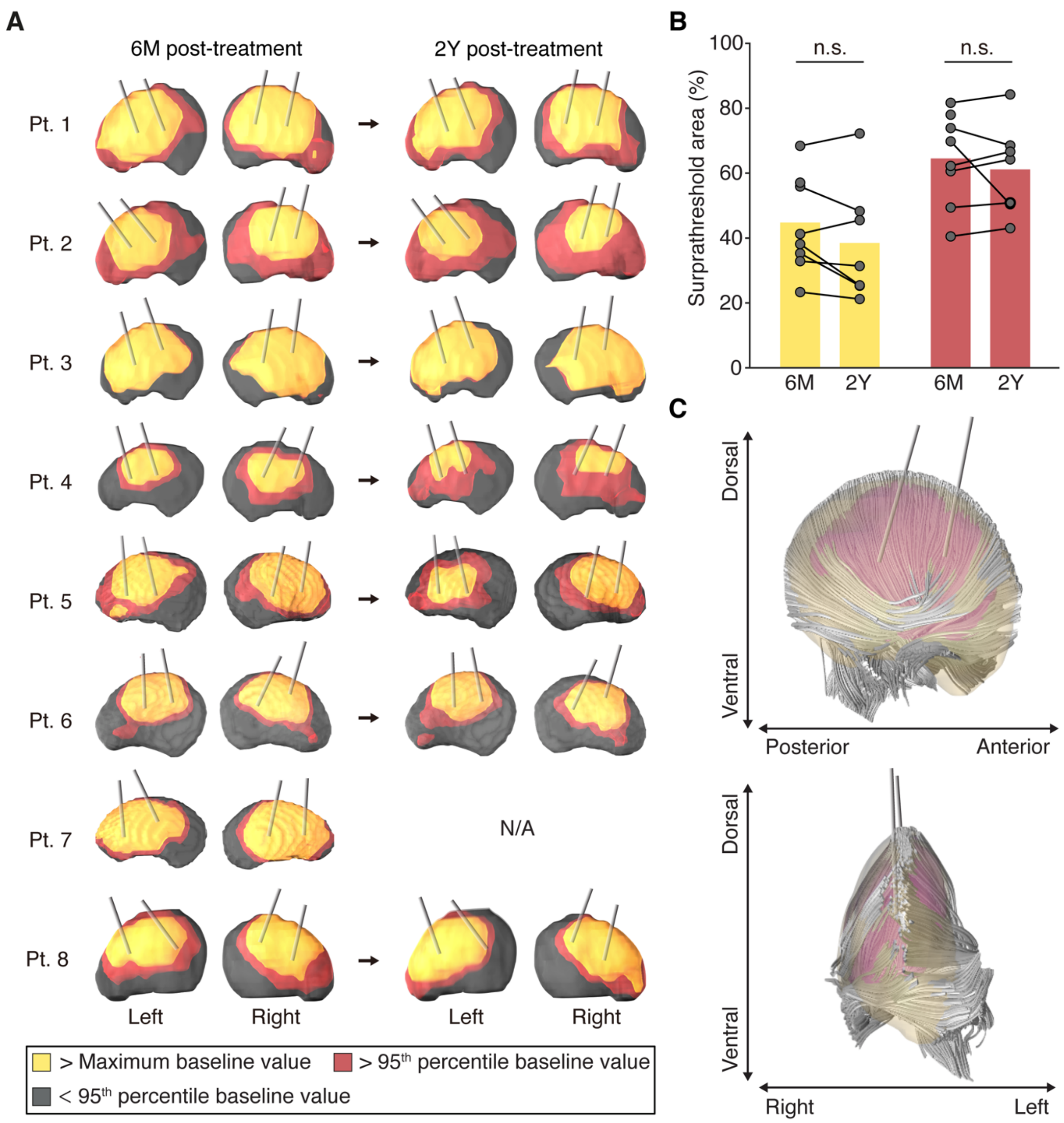

Supplementary Figure 5. 3D model of transduced areas in the putamen reconstructed from the fused structural MR and FMT-PET images. (A) 3D model of transduced areas in the putamen. The transduced area was broadly distributed from the locations of the vector-delivered needle tracks, and the area has been sustained for 2 years. The putaminal areas that exceeded the maximum baseline value and the 95th percentile baseline value and that fell below the 95th percentile baseline value are represented as yellow-, red-, and gray-colored areas, respectively. The two gray bars represent the needles for the vector injection. All putamen models are displayed as the lateral view. **(B)** The mean volume of the transduced

area across patients at 6M and 2Y post-treatment. Yellow- and red-colored bars represent the mean area that exceeds the maximum baseline value and the 95th percentile baseline value, respectively. **(C)** 3D model of the transduced area overlaid with the white matter tracts. It shows that the vector-containing solution diffused along the white matter tracts within the putamen. It is a representative example from Pt. 3 at 6M post-treatment. Silver-colored fibers represent the white matter tracts at baseline. The pink-colored area represents the area exceeding the maximum baseline value. The orange-colored area represents the entire putaminal area. The two gray bars represent the needles for the vector injection.

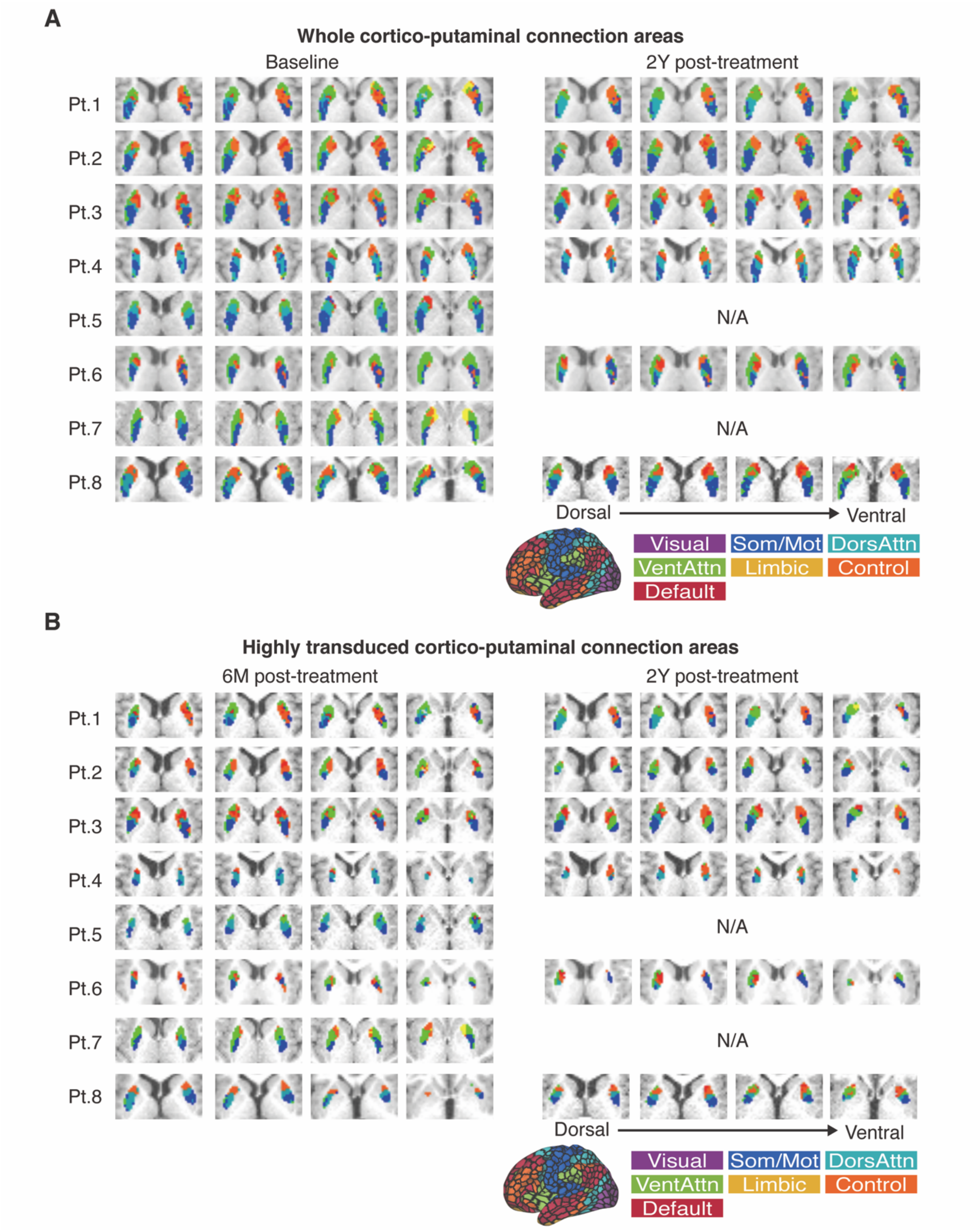

Supplementary Figure 6. Whole and highly transduced cortico-putaminal connection areas of seven cortical networks. (A) Whole cortico-putaminal connection areas of seven cortical networks at baseline and 2Y post-treatment. The proportion of the cortico-putaminal connection areas has been sustained for 2 years, and it was similar across all patients. Cortico-

putaminal connection areas were constructed by structural connectivity-based parcellation between the putaminal area and the 1000 cortical ROIs of seven networks. The connection area was segmented by different colors based on seven cortical networks. Som/Mot: somato/motor network; DorsAttn: dorsal attention network; VentAttn: ventral attention network; Control: frontoparietal control network. **(B)** Highly transduced cortico-putaminal connection areas of seven cortical networks at baseline and 2Y post-treatment (see Materials and methods and Fig. 3 for details).

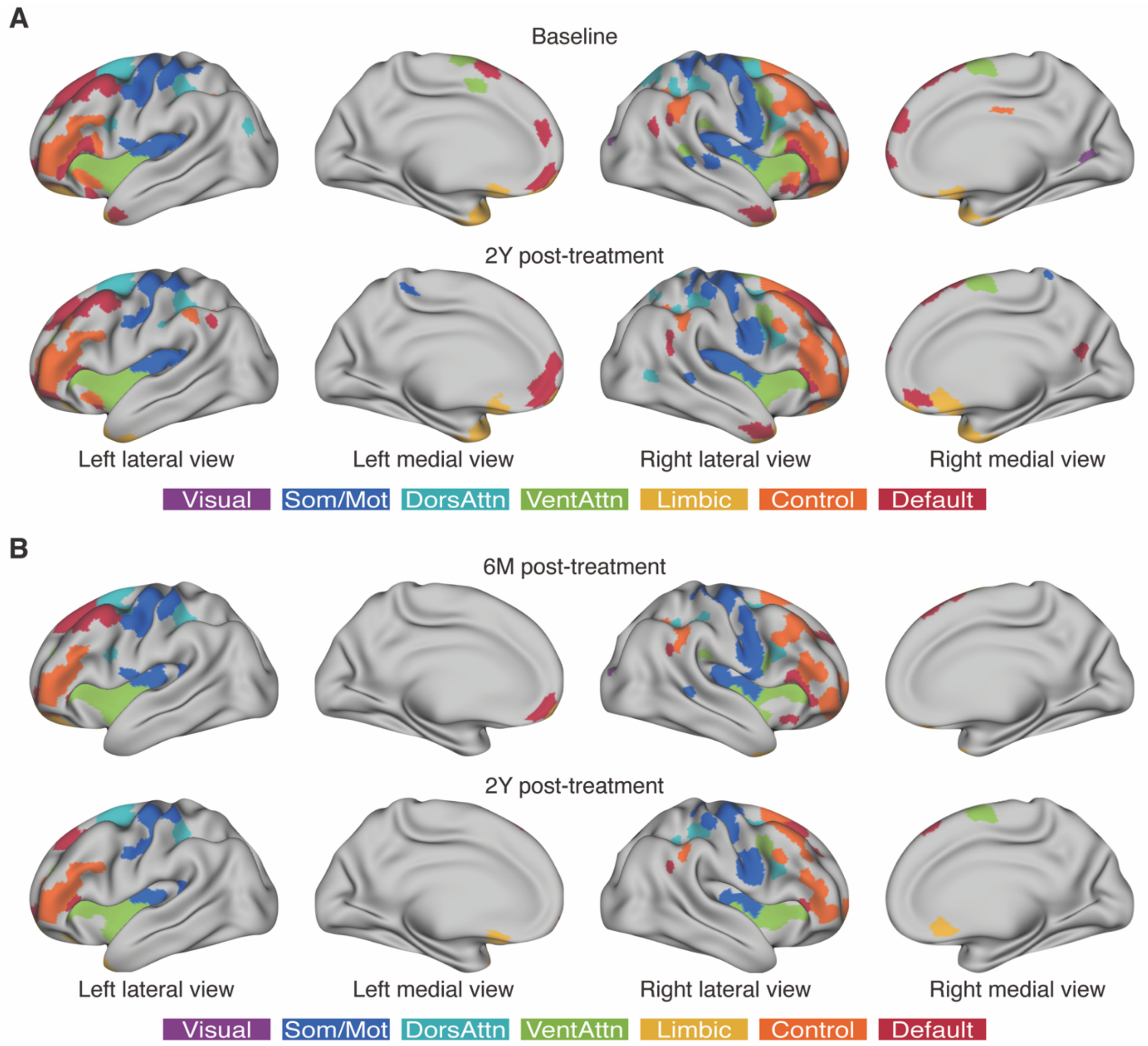

Supplementary Figure 7. Cortical areas that connected to the putaminal area across patients. (A) The 1000 cortical areas of the seven networks that dominantly connects to the entire putaminal areas at baseline and 2Y post-treatment. It indicates the putamen connects to all cortical networks except the visual network. **(B)** The 1000 cortical areas of the seven networks that dominantly connects to the highly transduced putaminal areas. It reveals that specific cortical signals from the frontoparietal control, somato/motor, dorsal attention, and a part of ventral attention networks mainly receive dopaminergic modulation.

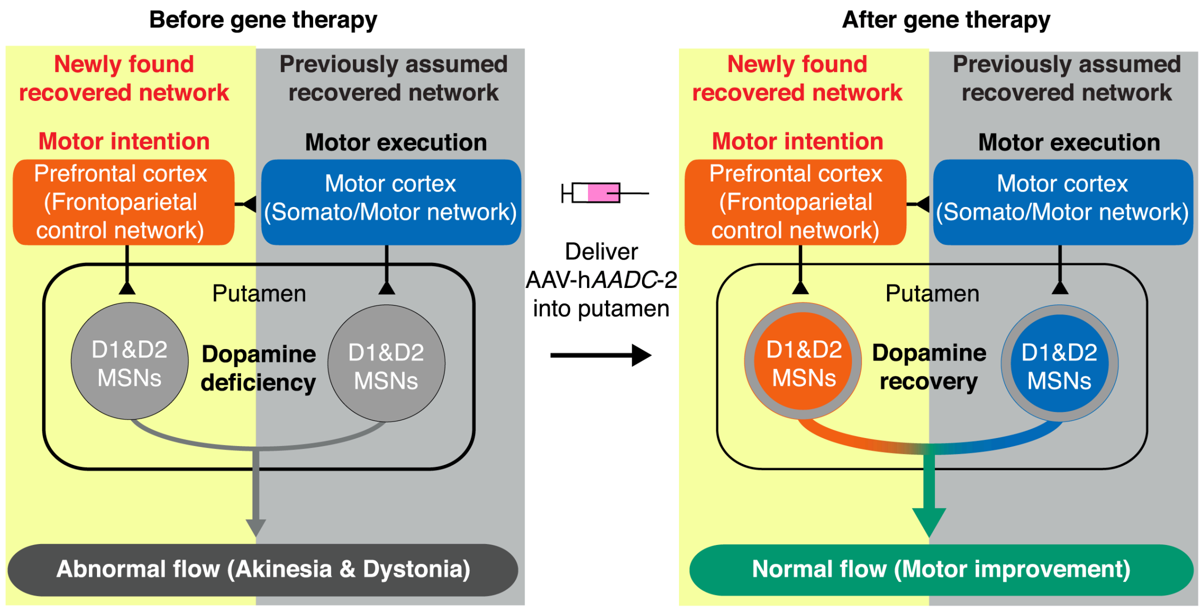

Supplementary Figure 8. Schematic diagram of the AADC gene therapy for motor improvement. The loss of dopamine in AADC deficiency causes excessive inhibition of cortical activity by increasing the GABAergic pallidal output to the thalamus (McGregor and Nelson, 2019), which results in akinesia and dystonia (Battistella and Simonyan, 2019). The AAV vectors infused into the putamen transduce mainly medium spiny neurons (MSNs) (Sehara et al., 2017). The broad transduction of the putamen leads to the recovery of glutaminergic inputs on the striatal medium spiny output neurons from pyramidal cortico-striatal neurons in the prefrontal cortex for motor intention and the motor cortex for motor execution (Jahanshahi et al., 2015; Benarroch, 2016).

Supplementary Table 1. Demographic features of patients

Patient ID	Age (Years)	Sex	Clinical manifestation	Severity	Mutations
1	15	Male	Prolonged bed rest, oculogyric crisis, dystonia, ptyalism, dyshidrosis	Severe	c.329C>A p.(Ala110Glu) unknown
2	12	Female	Prolonged bed rest, oculogyric crisis, dystonia, ptyalism, dyshidrosis	Severe	c.329C>A p.(Ala110Glu) unknown
3	5	Female	Motor development with support from oral medicine, oculogyric crisis	Moderate	c.315G>C, p.(Trp105Cys), c.385C>T p.(Pro129Ser)
4	19	Male	Prolonged bed rest, oculogyric crisis, dystonia, ptyalism, dyshidrosis, epilepsy	Severe	c.1106A>G p.(Try369Cys) IVS6+4A>T
5	10	Male	Prolonged bed rest, oculogyric crisis, dystonia, ptyalism	Severe	IVS6+4A>T IVS6+4A>T
6	4	Male	Prolonged bed rest, oculogyric crisis, dystonia	Severe	c.236A>G p.(Tyr79Cys) c.755A>G p.(Asp252Gly)
7	7	Female	Prolonged bed rest, oculogyric crisis, dystonia, ptyalism	Severe	c.236A>G (p.P87L). c.799T>C (p. W267R)
8	4	Male	Prolonged bed rest, oculogyric crisis, dystonia,	Severe	c.714+4A>T IVS6+4A>T

Supplementary Table 2. Significant volume of cortico-putaminal connection area of each structural region across patients

Structural regions (6M)	*t*-value (N = 8)	CI	*P* value
PFC	4.53	3.84–12.24	0.0054
PMC	8.71	7.56–13.19	0.0004
M1	4.93	2.44–6.96	0.0051
**Structural regions (2Y)**	***t*-value (N = 6)**	**CI**	***P* value**
PFC	5.75	5.30–13.87	0.0066
PMC	7.11	4.64–9.88	0.0036

Supplementary Table 3. Significant volume of cortico-putaminal connection area of each cortical network across patients

**Networks (6M)**	***t*-value (N = 8)**	**CI**	***P* value**
Frontoparietal control network	4.80	3.35–9.86	0.012
Somato/Motor network	6.20	5.50–12.28	0.0028
Dorsal attention network	4.67	3.86–11.79	0.012
**Networks (2Y)**	***t*-value (N = 6)**	**CI**	***P* value**
Frontoparietal control network	5.99	4.13–10.34	0.013
Somato/Motor network	4.18	2.73–11.40	0.043
Ventral attention network	4.28	2.54–10.17	0.047

Supplementary Table 4. Comparison of the volume of the prefrontal cortico-putaminal connection area of each network

Networks (6M)	*t*-value (N = 8)	CI	*P* value
Frontoparietal control network vs. Ventral attention network	4.30	2.34–8.05	0.011
Frontoparietal control network vs. Limbic network	3.89	1.88–7.69	0.012
Frontoparietal control network vs. Default network	3.20	0.85–5.64	0.015
**Networks (2Y)**	***t*-value (N = 6)**	**CI**	***P* value**
Frontoparietal control network vs. Ventral attention network	3.92	2.08–9.96	0.011
Frontoparietal control network vs. Limbic network	5.09	3.20–9.71	0.011
Frontoparietal control network vs. Default network	5.05	2.43–7.47	0.0078

Additional References

McGregor MM, Nelson AB. Circuit mechanisms of Parkinson's disease. *Neuron*. 2019;101(6):1042-1056.

Battistella G, Simonyan K. Top-down alteration of functional connectivity within the sensorimotor network in focal dystonia. *Neurology*. 2019;92(16):e1843-e1851.

Sehara Y, Fujimoto K, Ikeguchi K, et al. Persistent expression of dopamine-synthesizing enzymes 15 Years after gene transfer in a primate model of Parkinson's disease. *Hum Gene Ther Clin Dev*. 2017;28(2):74-79.

Jahanshahi M, Obeso I, Rothwell JC, Obeso JA. A fronto-striato-subthalamic-pallidal network for goal-directed and habitual inhibition. *Nat Rev Neurosci*. 2015;16(12):719-732.

Benarroch EE. Intrinsic circuits of the striatum. *Neurology*. 2016;86(16):1531-1542.
